# Discovery of Novel Drug Candidates for Alzheimer’s Disease by Molecular Network Modeling

**DOI:** 10.3389/fnagi.2022.850217

**Published:** 2022-04-15

**Authors:** Jiaxin Zhou, Qingyong Li, Wensi Wu, Xiaojun Zhang, Zhiyi Zuo, Yanan Lu, Huiying Zhao, Zhi Wang

**Affiliations:** ^1^Department of Anesthesiology, Sun Yat-sen Memorial Hospital, Sun Yat-sen University, Guangzhou, China; ^2^Medical Research Center, Sun Yat-sen Memorial Hospital, Guangzhou, China; ^3^Department of Anesthesiology, University of Virginia, Charlottesville, VA, United States

**Keywords:** Alzheimer’s disease, transcriptomic analysis, co-expressed modules, drug repurpose, aging

## Abstract

To identify the molecular mechanisms and novel therapeutic agents of late-onset Alzheimer’s disease (AD), we performed integrative network analysis using multiple transcriptomic profiles of human brains. With the hypothesis that AD pathology involves the whole cerebrum, we first identified co-expressed modules across multiple cerebral regions of the aging human brain. Among them, two modules (M3 and M8) consisting of 1,429 protein-coding genes were significantly enriched with AD-correlated genes. Differential expression analysis of microarray, bulk RNA-sequencing (RNA-seq) data revealed the dysregulation of M3 and M8 across different cerebral regions in both normal aging and AD. The cell-type enrichment analysis and differential expression analysis at the single-cell resolution indicated the extensive neuronal vulnerability in AD pathogenesis. Transcriptomic-based drug screening from Connectivity Map proposed Gly-His-Lys acetate salt (GHK) as a potential drug candidate that could probably restore the dysregulated genes of the M3 and M8 network. Pretreatment with GHK showed a neuroprotective effect against amyloid-beta-induced injury in differentiated human neuron-like SH-SY5Y cells. Taken together, our findings uncover a dysregulated network disrupted across multiple cerebral regions in AD and propose pretreatment with GHK as a novel neuroprotective strategy against AD.

## Introduction

Alzheimer’s disease (AD) is a slowly progressive, incurable neurodegenerative disease, characterized by cognitive impairment and neuropsychiatric symptoms (NPS) such as olfactory dysfunction, anxiety, depression, sleep disruption (Lyketsos et al., 2011; Eikelboom et al., 2021). AD is the most common cause of dementia in the world, accounting for around two-thirds of people living with dementia globally (Scheltens et al., 2016). Previous studies have already verified that aging is the greatest risk factor for AD (Brookmeyer et al., 2007; Hebert et al., 2013). With the acceleration of global population aging, AD has become a significant public health issue that needs to be resolved urgently (2021 Alzheimer’s disease facts and figures, 2021). However, the mainstream therapeutic medications show little effect on slowing down or stopping the progression of AD (Joe and Ringman, 2019). Thus, there is an urgent need to develop effective anti-AD drugs.

With the pathological features of amyloid-beta (Aβ) plaque and neurofibrillary tangle (NFT) in the human AD brain (Scheltens et al., 2016), AD is widely believed to be triggered by the production and accumulation of Aβ and hyperphosphorylated tau (p-tau) (Viola and Klein, 2015; Congdon and Sigurdsson, 2018). Based on the Aβ and p-tau hypothesis, transgenic mouse models overexpressing mutant APP and PSEN1 or MAPT (Holcomb et al., 1998; Oddo et al., 2003) are universally used for pathological mechanism research and preliminary medicines screening (Bilkei-Gorzo, 2014; Puzzo et al., 2015). However, although anti-Aβ antibodies show a promising neuroprotective effect on transgenic rodent models, 33 drugs targeting Aβ have failed to slow cognitive decline in phase 3 clinical trials (Ayton and Bush, 2021). Similarly, tau-based clinical trials have not yet produced positive results (Congdon and Sigurdsson, 2018). This is possibly because our knowledge of AD pathology is mostly based on the effects of some familial rare gene mutations, which are responsible for early-onset AD (EOAD) accounting for only 5% of the total AD cases (Drummond and Wisniewski, 2017). Meanwhile, current transgenic mouse models do not display the whole characteristic pathology of AD, such as aging and massive neuronal loss. Therefore, it is reasonable to think that late-onset AD (LOAD), accounting for the majority of the AD population, may be involved with other molecular processes disruption (Pimenova et al., 2018) and that it is necessary to take LOAD-correlated genes into account when determining the underlying mechanisms of AD.

Another problem that needs to be resolved concerning AD is the pathologically vulnerable brain regions. The majority of research often focuses on cognitive-related brain regions such as the hippocampus (HIP) and the prefrontal cortex (PFC). However, diverse NPS imply that AD might be associated with abnormalities in different brain areas, including the frontal cortex, HIP, entorhinal cortex, amygdala, etc. (Chen et al., 2021). Neuroimaging techniques such as MRI, functional MRI, Aβ PET, glucose metabolism PET, and diffusion tensor imaging also indicate many AD-related regions such as the PFC, temporal cortex, cingulum, precuneus, entorhinal cortex (EC), hippocampal body, parahippocampal gyrus (PHG), amygdala, cingulum bundles, and corpus callosum (Márquez and Yassa, 2019; Wang et al., 2020). We, therefore, hypothesize that AD pathological changes involve the whole brain, but are not restricted to a certain brain region.

Some databases already collect AD-associated genes and variants from multiple resources including genome-wide association studies (GWAS) and other large-scale association studies, animal models, and scientific literature (Rappaport et al., 2017; Piñero et al., 2020). Gene network analysis provides a powerful approach to elucidate a comprehensive understanding of dysregulated molecular processes underlying disease, as opposed to traditional single-gene approaches (Parikshak et al., 2015). The application of co-expression network analysis has already identified dysregulated networks of AD in the human dorsolateral PFC (DLPFC) and HIP (Miller et al., 2013; Zhang et al., 2013). Beyond discovering the underlying mechanism of disease, network analysis also provides a new strategy for drug discovery or repurposing (Lamb et al., 2006; Iorio et al., 2010). Drugs are often screened by their ability to induce transcriptional responses, not by their binding affinity to specific proteins in a traditional way.

**FIGURE 1:**
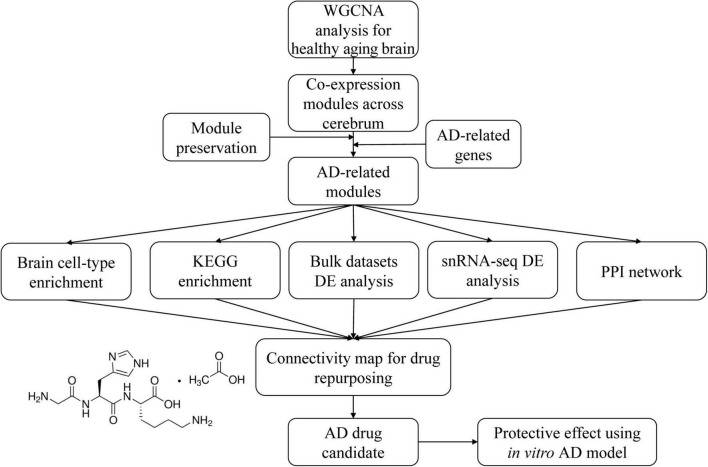
Schematic design on how to discover Alzheimer’s disease (AD)-related modules and anti-AD drug candidates.

In the present study, because NPS and neuroimaging results imply that multiple brain regions are involved in AD, we utilized a systematic approach to investigate the underlying dysregulated gene network across multiple brain regions and further screen potential agents against AD by targeting this network. The workflow of our experimental design is shown in Figure 1. We first constructed gene co-expression networks (modules) across different cerebral regions from normal aging individuals to identify the common physiological processes across the whole cerebrum. To identify modules relevant to AD, we collected AD-correlated genes from three datasets and reported two modules significantly enriched with AD-correlated genes. Functional enrichment and cell-type enrichment analysis revealed that the two modules were mainly involved in the pathway of synapse function and energy metabolism in neuronal cells. Differential expression (DE) analysis of microarray, bulk RNA sequencing (RNA-seq), and single-nucleus RNA-seq (snRNA-seq) data suggested the dysregulation of the two modules across multiple cerebral regions and extensive neuronal cell-types in normal aging and AD. Considering the differential expressed genes (DEGs) of the two modules as potential targets, we proposed Gly-His-Lys acetate salt (GHK) as a drug candidate that could probably restore the disordered DEGs toward health using the Connectivity map (CMap) dataset. The neuroprotective effect of GHK and its ability to reverse the dysregulated network was finally verified *in vitro*.

## Materials and Methods

### Data Collection and Processing

The public transcriptome datasets for this study were downloaded from gene expression omnibus (GEO),^1^ Genotype-Tissue Expression project version 8 (GTEx),^2^ and Synapse^3^ under corresponding accession numbers. The details of the datasets are presented in Supplementary Table 1. For the microarray data, gene-level expression was obtained by taking the maximum expression values of multiple probes mapping to the same gene. For the RNA-seq data, we filtered out lowly expressed genes by retaining the gene with at least 10 counts in at least half of the samples. Count data were then normalized using Trimmed Means of M values (TMM) of edgeR R package (Robinson and Oshlack, 2010) to adjust for sequencing library size difference. For the snRNA-seq data, the filtered count matrix was normalized by first running the quickCluster function of scran R package (Lun et al., 2016) with the block parameter set to the sample identity of each cell, then estimating size factors by using computeSumFactors function with the parameter min. mean = 0.1 and cluster parameter set to clusters. The LogNormCounts function of scater R package (McCarthy et al., 2017) was subsequently implemented to compute normalized expression values for each cell using size factors identified by computeSumFactors function. Only protein-coding genes (human genome-version GRCh37) were saved for subsequent analysis.

### Construction of Modules Across Multiple Cerebral Regions

The RNA-Seq transcriptional profile of 12 brain regions was downloaded from GTEx (GTEx Consortium, 2013). We selected samples with the following filtering criteria: (a) RNA integrity number (RIN)>5, (b) mapping rate>0.7, (c) exonic mapping rate>0.7, (d) age > 60. Principal component analysis (PCA) analysis was performed using the prcomp function with scaling. The transcriptomic data of each brain region was corrected by applying linear mixed modeling for each gene using the fitVarPartModel function of the variancePartition R package (Hoffman and Schadt, 2016). The normalized data was used as input data for co-expression modules construction using weighted gene co-expression network analysis (WGCNA) R package (Langfelder and Horvath, 2008) with the following parameters: soft threshold power (β) = 6 (chosen based on the scale-free topology model fit plot *r*^2^ > 0.8), type = “signed,” corType = “bicor,” mergeCutHeight = 0.2, minModuleSize = 50.

### Preservation of Co-expression Modules in Other Datasets

The processed microarray data of multiple human brain regions were downloaded from GEO under the accession number GSE60862 (Trabzuni et al., 2013). Samples of the cerebellar cortex were excluded. Only individuals with age over 60 were retained. RNA-seq data of human blood tissue was downloaded from GTEx (GTEx Consortium, 2013). Likewise, we selected individuals with ages over 60. RNA-seq data of normal aging mouse cortex and HIP (18 months old) was downloaded from GSE168137 (Forner et al., 2021). Mouse genes were converted to human genes using biomaRt R package (Durinck et al., 2009) and only the “one-to-one” ortholog genes were saved. The modulePreservation function (Langfelder et al., 2011) of the WGCNA R package was used to calculate Zsummary values for each dataset.

### Spatiotemporal Analysis of Gene Set Expression

The expression data of 10 cerebral regions from GTEx were classified into three age groups: 20–39, 40–59 and 60–79. To assess the gene set activity of M3 and M8 for each sample, gene set variation analysis (GSVA) with ssGSEA method was implemented to calculate gene set enrichment scores using the GSVA R package (Hänzelmann et al., 2013). GSVA scores of different age groups were compared using the Mann-Whitney test with Benjamini-Hochberg correction.

### DE Analysis Across Multiple Cerebral Regions at the Tissue Level

Microarray datasets: The transcriptional profile of the DLPFC was downloaded from GSE33000 (Narayanan et al., 2014). Three independent transcriptional profiles of the EC were downloaded from GSE118553 (Patel et al., 2019), GSE48350 (Cribbs et al., 2012), and GSE5281 (Liang et al., 2008). Three independent transcriptional profiles of the HIP were downloaded from GSE29378 (Miller et al., 2013), GSE48350 (Cribbs et al., 2012), and GSE5281 (Liang et al., 2008). Different microarray datasets of the same brain region were merged using the ComBat algorithm of sva R package (Johnson et al., 2007). The efficacy of batch effect removal was examined by PCA. Differential expression (DE) analysis for each brain region was performed using the limma R package (Ritchie et al., 2015) with the age of death and sex as covariates. Genes with *q* < 0.05 (*q*-values were determined by *p*-values corrected with the Benjamini-Hochberg method) were defined as significant genes.

RNA sequencing dataset: The read counts of four brain regions: frontal pole (FP), superior temporal gyrus (STG), PHG, and inferior frontal gyrus (IFG) were obtained from Synapse under Synapse ID syn7391749 (Wang et al., 2018a). We removed samples from Asian and unknown ancestry because of too few samples. The samples were assigned with a clinical dementia rating (CDR) score “0” as control and “2–5” as AD group. DE analysis was performed using the DEseq2 R package (Love et al., 2014), with batch, sex, race, age of death, RIN, postmortem interval (PMI), and recombinant RNA (rRNA)-rate as covariates. Genes with *q* < 0.05 were defined as significant genes.

### Cell-Type Enrichment Analysis

Two snRNA-seq datasets were downloaded from Synapse under Synapse ID syn18485175 (Mathys et al., 2019) and syn21788402 (Leng et al., 2021). On the basis of cell-types identified by Mathys et al., marker genes for each cell-type against the rest of cell-types were detected using the FindAllMarkers function of the Seurat R package (Satija et al., 2015). Marker genes for each cell-type were selected based on the cutoff criteria of absolute log2 fold change (logFC) > 1, *q* < 0.05 and percentage of expressed cells >30%. For cell-type enrichment analysis, a one-tailed Fisher’s exact test (FET) with Benjamini-Hochberg correction was performed for M3 and M8 against the cell-type marker gene lists.

### Gene Set Modular Score Calculation and DE Analysis at the Single-Cell Level

The gene set expression score of M3 and M8 for each neuronal cell was defined as the average relative expression of the gene set of M3 and M8 minus the average relative expression of a control gene set, which was calculated using the AddModuleScore function (Tirosh et al., 2016) of Seurat R package. Neuronal subtypes containing fewer than 500 cells were excluded. DE analysis for each cell type was performed between AD cells and control cells using the MAST R package (Finak et al., 2015). Only genes with a percentage of expressed cells >20% were used for DE analysis. We fit a hurdle model modeling the disease condition and gene detection rate (cngeneson) to adjust for the cngeneson, and then ran a likelihood ratio test (LRT) to identify genes differentially expressed due to the AD.

### Kyoto Encyclopedia for Genes and Genomes Enrichment Analysis

Kyoto Encyclopedia of Genes and Genomes (KEGG) enrichment analysis and network visualization were conducted on the Metascape online platform (Zhou et al., 2019).^4^ Only terms with *p* < 0.01, minimum network size >3, maximum network size <500, and enrichment factor >1.5 were considered as significant. The enrichment network was created by representing each significant term as a node and connecting pairs of nodes with Kappa similarities >0.3.

### Protein-Protein Interaction Network Construction

Protein-protein interaction (PPI) network of DEGs was constructed on the STRING online tool (Szklarczyk et al., 2019)^5^ and visualized on the Cytoscape software (Shannon et al., 2003). The network was created by representing each gene as a node and connecting pairs of nodes with confidence >0.5.

### Drug Repurposing Analysis

For drug repurposing using the CMap database (Lamb et al., 2006), a preprocessing step was undertaken to convert DEGs from gene symbols to the required HG-U133A (GPL96) probes. We uploaded upregulated and downregulated probes simultaneously for CMap query (build02).^6^ Drugs were ranked by enrichment score and percent non-null after discarding drugs with *p* > 0.05 and number of instances <2. Negative enrichment values of the drug repurposing list meant that drugs could possibly reverse the dysregulated genes.

Cell files from the PC3 cell line treated with GHK and matched vehicle pairs were downloaded from CMap and pre-processed using the Affymetrix RMA algorithm of the affy R package (Gautier et al., 2004). Gene-level expression was obtained by taking the maximum of the expression values of multiple probes mapping to the same gene. DE analysis was performed using the limma R package (Ritchie et al., 2015). Gene set enrichment analysis (GSEA) (Subramanian et al., 2005) was applied for the pre-ranked gene list using the clusterProfiler R package (Yu et al., 2012). Significant GSEA pathways were selected with *q* < 0.05.

### Reagents

Aβ_25–35_ peptide and all primer pairs were synthesized by Sangon Biotech Co., Ltd. (Guangzhou, China). GHK and All-trans retinoic acid were bought from Sigma-Aldrich (St. Louis, MO, United States). DMEM/F12 medium and L-glutamine were purchased from Gibco (California, United States). Neurobasal medium and B-27 were obtained from Gibco (Invitrogen, United States). Fetal bovine serum was purchased from Gibco (Australia). Reverse Transcription Kit, and SYBR Green PCR Kit were provided by Yeasen (Shanghai, China), and LDH Release Assay Kit was purchased from Beyotime (Shanghai, China).

### Amyloid-Beta and Gly-His-Lys Acetate Salt Preparation

Aβ_25–35_ peptide was dissolved in sterile distilled water at a concentration of 2 mM stock solution and incubated in a capped vial at 37°C for 7 days to allow the formation of the aggregated form, which was then stored frozen at −20°C before use. GHK was dissolved in sterile distilled water at a concentration of 100 μM stock solution and was stored frozen at −20°C before use.

### Cell Culture and Treatment

Human neuroblastoma SH-SY5Y cells were cultured as described previously (Lin et al., 2011). Undifferentiated SH-SY5Y cells were first cultured in DMEM/F12 medium supplemented with 10% fetal bovine serum at a condition of 37^°^C and 5% CO_2_ atmosphere. Cells were seeded at an appropriate density (5 × 10^4^ cells/mL) in 24-well plates. One day after seeding, the culture medium was changed into a neurobasal medium, supplemented with B-27, L-glutamine (500 μM), and all-trans retinoic acid (RA, 10 μM) for 3 days to induce cells to fully differentiate into human neuron-like cells. These cells were then used for biochemical and molecular experiments. Differentiated SH-SY5Y cells with different treatments were used: (a) control (cells treated with vehicle for 24 h); (b) Aβ_25–35_ (cells treated with Aβ_25–35_ for 24 h); (c) GHK (cells treated with GHK for 24 h); and (d) GHK + Aβ_25–35_ (cells pretreated with GHK for 6 h before the addition of Aβ_25–35_ for another 24 h).

### Lactate Dehydrogenase Release Assay

Lactate dehydrogenase (LDH) activity was determined using an LDH cytotoxicity detection kit as done previously (Lin et al., 2011). Briefly, the incubation medium harvested from the 24-well plates was centrifuged at 13,000 rpm for 10 min at 4°C and 120 μL of the cell-free supernatant was transferred to 96-well plates for extracellular LDH activity measurement. After the removal of the incubation medium, the remaining cells were washed twice with phosphate-buffered saline (PBS). A 1% triton X-100 lysing solution was then added to the cells and incubated for 15 min. The cell lysates were also centrifuged at 13,000 rpm for 10 min at 4°C and 120 μL of the supernatant was transferred to 96-well plates for intracellular LDH activity measurement. The activity of extracellular and intracellular LDH was determined by a colorimetric assay with the absorbance at a wavelength of 490 nm according to the manufacturer’s instructions. The background absorbance from the culture media and 1% triton X-100 lysing solution was subtracted from extracellular and intracellular LDH absorbance measurements, respectively. The ratio of released LDH (extracellular) vs. total LDH (extracellular + intracellular) was calculated as an indicator of cell injury.

### RNA Isolation and Quantitative Polymerase Chain Reaction Assay

The expression levels of hub genes were quantified by Real-time quantitative polymerase chain reaction (qPCR) following the methods described previously (Wang et al., 2018b). Briefly, the total RNA was extracted from cells using a TRIzol reagent. A total of 0.5 μg of the total RNA was used for reverse transcription using a Reverse Transcription Kit according to the manufacturer’s instructions. Subsequently, qPCR was carried out using SYBR Green PCR Kit and performed on 480 LightCycler (Roche). The mRNA level of GAPDH was used as an internal control. Sequences of primer pairs used in reverse transcription are available in Supplementary Table 2.

### Statistical Analysis

Bioinformatics analyses and graphical visualizations were performed on R 4.0.2^7^ and online tools. Experimental statistical analyses and graphical visualizations were performed using GraphPad Prism. Experimental results were presented as means ± SEM. Each experiment was repeated at least three times. Experimental statistical analyses were performed using ANOVA, followed by Dunnet’s *post hoc* analysis. *P* values were defined as follows: *p < 0.05, ^**^p < 0.01, ^***^p < 0.001, ^*⁣*⁣**^p < 0.0001.

## Results

### Construction of Co-expression Modules Across Multiple Cerebral Regions

With the hypothesis that the functional pathways throughout the brain are disrupted in AD, we used a large-scale transcriptomic dataset of different brain regions from cognitively normal individuals (GTEx Consortium, 2013) to identify gene co-expression modules. The expression data comprised two cerebellar regions: cerebellar hemisphere and cerebellum, and 10 cerebral regions: amygdala, anterior cingulate cortex, caudate, cortex, frontal cortex, HIP, hypothalamus, nucleus accumbens, putamen, and substantia nigra. Since age is the greatest risk factor for the development of AD, we selected individuals with age over 60 to construct co-expression modules for a normal aging brain. We first performed PCA to visually evaluate the differences in gene expression patterns between different brain regions. As shown in Supplementary Figure 1A, samples from the cerebellum were clearly separated from the rest of the cerebral regions, indicating that the cerebellum had a different gene expression pattern from the cerebrum. In addition, clinical symptoms and transcriptomic evidence imply that the cerebellum is relatively more rarely affected in AD pathogenesis (Andersen et al., 2012; Tiwari et al., 2015). We, therefore, discarded samples from the cerebellum in the subsequent analysis. To determine the effect of covariates on gene expression, we calculated the proportion of variance in RNA expression explained by covariates including age, gender, RIN, center, and DTHHRDY (Hardy Scale) for each cerebral region (Supplementary Figures 1B–K). Since age, gender, and center showed little effect (less than 10% variance explained by covariates) on most genes, the expression data of each cerebral region was corrected for RIN and DTHHRDY by fitting linear mixed modeling. WGCNA algorithm (Langfelder and Horvath, 2008) was next applied to the corrected expression data across 10 cerebral regions and identified 19 co-expressed modules with sizes ranging from 68 genes to 3,192 genes, while 3,383 genes were not clustered into any module (Supplementary Figures 2A,B).

**FIGURE 2:**
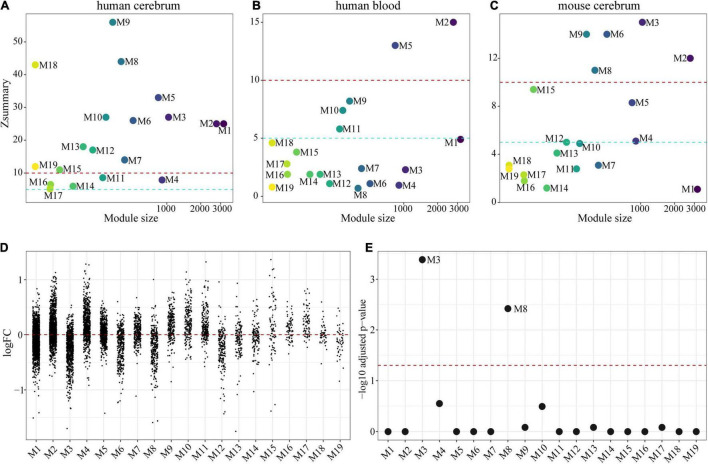
Preservation of co-expressed modules and identification of AD-related modules. **(A–C)** Preservation scores for 19 co-expressed modules in other expression datasets: **(A)** across multiple cerebral regions of aging human brains, **(B)** of human blood tissue, **(C)** across aging mouse cortex and hippocampus. Each dot represents a weighted gene co-expression network analysis (WGCNA) module. Dashed red and blue lines represent Zsummary = 10 and Zsummary = 5, respectively. **(D)** LogFC values of genes in each module between AD patients and controls. **(E)** Statistical significance of each module enriched with AD-correlated genes. *Q*-values were determined by FET *p*-values corrected with the Benjamini-Hocheberg method. Each dot represents a module and a dashed line means FET *q* = 0.05.

We then tried to validate whether these modules were reproducible in other transcriptomic datasets. For this purpose, we used another microarray dataset across multiple cerebral regions of aging human brains (Trabzuni et al., 2013), a transcriptomic profile from aging human blood tissue (GTEx Consortium, 2013), and a transcriptomic profile from aging mouse HIP and cortex (Forner et al., 2021). Here, Zsummary was used to evaluate module preservation (Langfelder et al., 2011) and we chose the following thresholds: Zsummary > 10 indicating strong module preservation, Zsummary > 5 indicating moderate module preservation, and Zsummary < 5 indicating poor module preservation. We observed that the co-expression modules were generally well preserved in another dataset of the human cerebrum (14 out of 19 modules with Zsummary > 10; 5 out of 19 modules with Zsummary > 5; Figure 2A), but poorly preserved in the blood tissue (2 out of 19 modules with Zsummary > 10; 3 out of 19 modules with Zsummary > 5; Figure 2B). As for module preservation across species, 5 out of 19 modules were well preserved (Zsummary > 10) and 4 out of 19 modules were moderately preserved (Zsummary > 5) in the mouse cerebrum dataset (Figure 2C). These results suggest that human brain has a different expression pattern from other tissues such as blood, and that mouse brain only mimics the human brain to a certain extent in a transcriptomic pattern.

To discover overall gene expression change for each module in AD pathogenesis, we performed DE analysis between AD cases and controls using a microarray dataset of the human DLPFC (De Jager et al., 2018). As shown in Figure 2D, 5 modules were generally downregulated and 8 were generally upregulated.

### Identification of Alzheimer’s Disease-Associated Co-expression Modules

Alzheimer’s disease-correlated genes were collected from a comprehensive review (Agrawal, 2017), as well as two human disease databases including MalaCards (Rappaport et al., 2017) and DisGeNet (Piñero et al., 2020). These databases contain genes and variants identified by GWAS and other large-scale association studies, animal models, and scientific literature. Totally, 323 protein-coding genes were retained as AD-correlated genes (Supplementary Table 3). By integrating co-expression modules with AD-correlated genes using one-tailed FET with Benjamini-Hochberg correction, we identified two modules, M3 [*q* = 4.14 × 10^–4^, odds ratio (OR) = 2.20] and M8 (*q* = 3.78 × 10^–3^, OR = 2.59), which were significantly enriched with AD-correlated genes (Figure 2E). These two modules contained 1,429 protein-coding genes in total.

**FIGURE 3:**
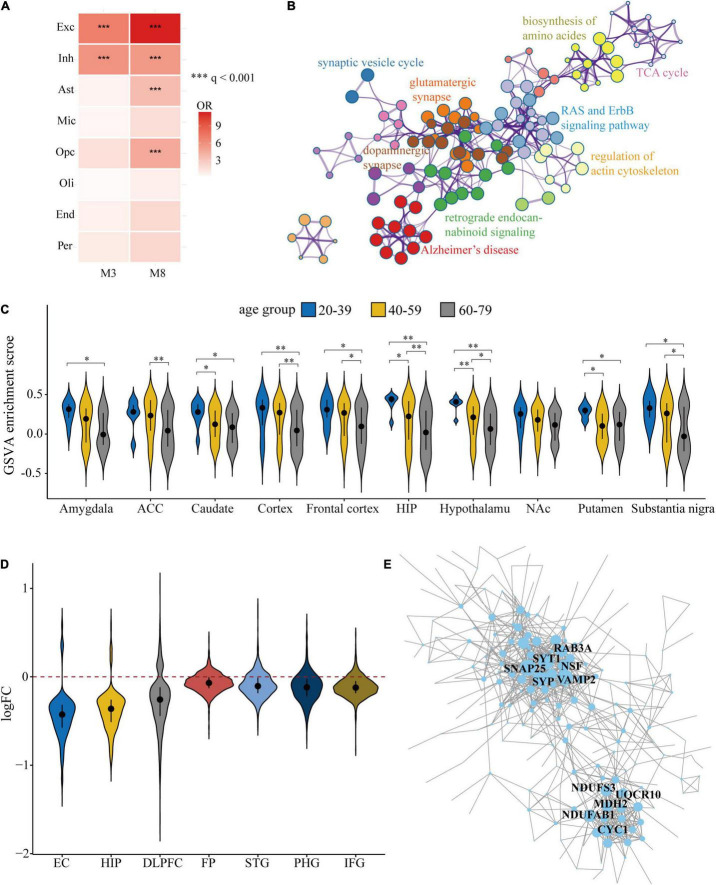
Functional enrichment and expression changes of M3 and M8 in normal aging and AD across multiple cerebral regions. **(A)** Brain cell-type enrichment for M3 and M8. The heatmap shows the one-tailed Fisher’s exact test (FET) results with BH correction for each brain cell type. Exc, excitatory neurons; Inh, inhibitory neurons; Ast, astrocytes; Mic, microglia; OPC, oligodendrocyte progenitor cell; Oli, oligodendrocytes; End, endothelial cells; Per, pericytes. **(B)** Kyoto encyclopedia of genes and genomes (KEGG) pathway enrichment analysis for M3 and M8. The enrichment network shows the intra-cluster and inter-cluster similarities of enriched KEGG terms. Color code represents the cluster annotations. **(C)** Comparison of sample-wise gene set enrichment scores of M3 and M8 in three different age groups of different cerebral regions. Kruskal-Wallis test with Benjamini-Hochberg correction was used to determine statistical significance. The black circle and bar represent median and quartiles (25th and 75th percentile), respectively. *q < 0.05, ^**^q < 0.01, ^***^q < 0.001. ACC, Anterior cingulate cortex; HIP, hippocampus; NAc, nucleus accumbens. **(D)** Violin plots of logFC values for genes of M3 and M8 between AD cases and controls in 7 cerebral regions. EC, entorhinal cortex; HIP, hippocampus; DLPFC, dorsolateral prefrontal cortex; PCG, postcentral gyrus; FP, frontal pole; STG, superior temporal gyrus; PHG, parahippocampal gyrus; IFG, inferior frontal gyrus. **(E)** PPI network of 345 DEGs with absolute logFC > 0.2 and *q* < 0.05 in at least 4 brain regions. The network was created by representing each gene as a node and connecting pairs of nodes with confidence >0.5. Node size is proportional to the degree. Hub genes with the highest degrees are labeled in black.

To annotate M3 and M8 for certain cell-type expressions, we took advantage of a published snRNA-seq dataset from human PFC (Mathys et al., 2019) to determine marker genes for each brain cell type. Marker gene lists of each cell type were then used to assess cell type enrichment for each module by one-tailed FET. As shown in Figure 3A, M3 and M8 were both significantly enriched for excitatory neurons (M3: *q* = 4.92 × 10^–26^; M8: *q* = 3.47 × 10^–24^) and inhibitory neurons (M3: *q* = 6.45 × 10^–15^; M8: *q* = 3.81 × 10^–7^). Together with the similar expression changes in AD pathogenesis, we merged the two modules for subsequent analysis.

We next conducted a KEGG pathway enrichment analysis to reveal a detailed biological function spectrum for M3 and M8. Functionally, M3 and M8 were highly enriched in pathways of synaptic vesicle cycle (*q* = 2.09 × 10^–11^), retrograde endocannabinoid signaling (*q* = 1.17 × 10^–10^), biosynthesis of amino acids (*q* = 3.09 × 10^–8^), and tricarboxylic acid (TCA) cycle (q = 2.19 × 10^–4^) (Figure 3B). These pathways are closely related to key biological processes in neurons, which is consistent with the results of cell-type enrichment analysis.

To understand the influence of aging (the strongest risk factor for AD) on the expression levels of M3 and M8, we divided cognitively normal individuals from GTEx into three age stages: 20–39, 40–59 and 60–79. GSVA was implemented for the gene set of M3 and M8 to yield single sample enrichment scores. As shown in Figure 3C, M3 and M8 tended to decrease with normal aging in most cerebral regions, which might relate to the age-dependent increase in susceptibility to AD.

To elucidate whether M3 and M8 were also consistently dysregulated in different AD brain regions, we collected expression data from 7 cerebral regions, including EC, HIP, DLPFC, FP, STG, PHG, and IFG. For HIP and EC, we implemented the ComBat algorithm (Leek et al., 2012) to merge the expression data from different microarray platforms and examined the efficacy of batch effect removal by PCA (Supplementary Figures 3A–D). DE analysis was then performed between the AD cases and controls for each brain region. We found that genes of M3 and M8 showed similar expression changes in different brain regions, most of which presented a downward trend (Figure 3D). DEGs of M3 and M8 were selected according to the criteria of absolute logFC > 0.2 and *q* < 0.05 in at least four brain regions. Among 1,429 genes of M3 and M8, 345 co-expression genes were defined as DEGs, most of which were downregulated (335 genes) (Supplementary Table 4). We next constructed the curated PPI network for DEGs (Figure 3E) and identified the first 10 hub genes including SNAP25 (synaptosome associated protein 25), SYP (synaptophysin), NSF (N-ethylmaleimide sensitive factor, vesicle fusing ATPase), SYT1 (synaptotagmin 1), NDUFAB1 (NADH: ubiquinone oxidoreductase subunit AB1), VAMP2 (vesicle associated membrane protein 2), CYC1 (cytochrome c1), NDUFS3 (NADH:ubiquinone oxidoreductase core subunit S3), MDH2 (malate dehydrogenase 2) and RAB3A (member RAS oncogene family). These hub genes were critically involved in synaptic activity and oxidative phosphorylation.

**FIGURE 4:**
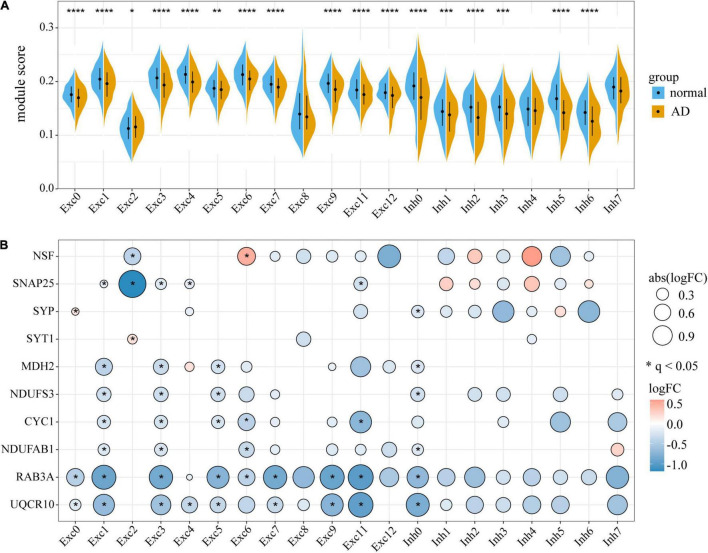
Expression change of M3 and M8 in AD at single-cell resolution. **(A)** Comparison of gene expression scores of M3 and M8 between AD cases and controls in different neuronal subtypes of the prefrontal cortex (PFC). Mann-Whitney test with Benjamini-Hochberg correction was used to determine statistical significance. The black circle and bar indicate median and quartiles (25th and 75th percentile), respectively. *q < 0.05, **q < 0.01, ***q < 0.001, ****q < 0.0001. **(B)** Differential expression analysis of hub genes between AD patients and controls in different neuronal subtypes. Blue and red colors indicate downregulation and upregulation respectively. Only genes with absolute LogFC > 0.14 are shown. *q < 0.05. Exc, excitatory neuron; Inh, inhibitory neuron.

The obvious restriction of bulk tissue transcriptome profiling is that it only represents an average of gene expression across diverse cell types. SnRNA-Seq provides a powerful approach for identifying cell-specific gene expression changes in brain diseases. To investigate whether the expression changes of M3 and M8 were also present at the single-cell level, we calculated the gene set expression scores of M3 and M8 for different neuronal subtypes of two independent AD snRNA-Seq datasets (Mathys et al., 2019; Leng et al., 2021). It was apparent that M3 and M8 were downregulated in most excitatory and inhibitory neuronal subtypes of AD PFC (Figure 4A). Similar expression downregulation was observed in most neuronal subtypes of AD superior frontal gyrus (SFG) (Supplementary Figure 4A) and EC (Supplementary Figure 4B). Notably, we observed that the expression levels M3 and M8 were decreased with increased Braak stage which marks the severity of tau-based NFT pathology. DE analysis in a cell-type specific manner also revealed that hub genes identified by the PPI network were mostly downregulated in different neuronal cell-types of AD PFC (Figure 4B), but rarely in non-neuronal cell-types, basically because of low detection of genes in non-neuronal cell populations (data not shown).

## Gly-His-Lys Acetate Salt Was Predicted to be an Anti-AD Drug Candidate by Molecular Network Modeling

**FIGURE 5:**
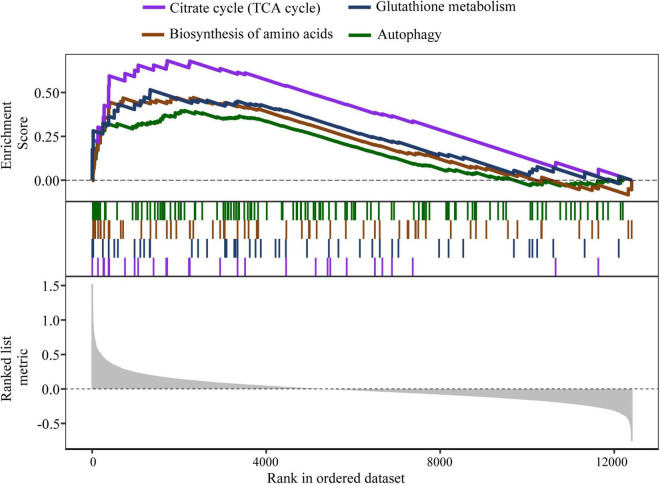
Representative GSEA enrichment pathways of GHK-induced transcriptional responses. The pathways shown in the picture are citrate cycle (TCA cycle) (hsa00020); glutathione metabolism (hsa00480); biosynthesis of amino acids (hsa01230); and autophagy-animal (hsa04140).

We hypothesized that DEGs from M3 and M8 represented a potential target for treating AD and therefore aimed to identify drug candidates whose effect on gene expression could restore the dysregulation of DEGs toward health. CMap (Lamb et al., 2006) is a library containing genome-wide transcriptional expression profiles from human cell lines treated with over 1,300 small molecules. We took advantage of this database to explore new indications of existing drugs that could reverse the DEGs and found that Gly-His-Lys acetate salt (GHK) was on the top of the drug list (enrichment score = −0.853). To screen the potential effects of GHK on cells, we carried out DE analysis and GSEA (Subramanian et al., 2005) to identify GHK-induced transcriptomic change. As indicated in Figure 5, treatment with GHK affected multiple signaling pathways, including citrate cycle (*q* = 2.88 × 10^–3^, NES = 2.21), biosynthesis of amino acids (*q* = 0.028, NES = 1.78), glutathione metabolism (*q* = 0.029, NES = 1.82), and autophagy (*q* = 0.018, NES = 1.67). A more complete description of the pathways induced by GHK was shown in Supplementary Table 5.

**FIGURE 6:**
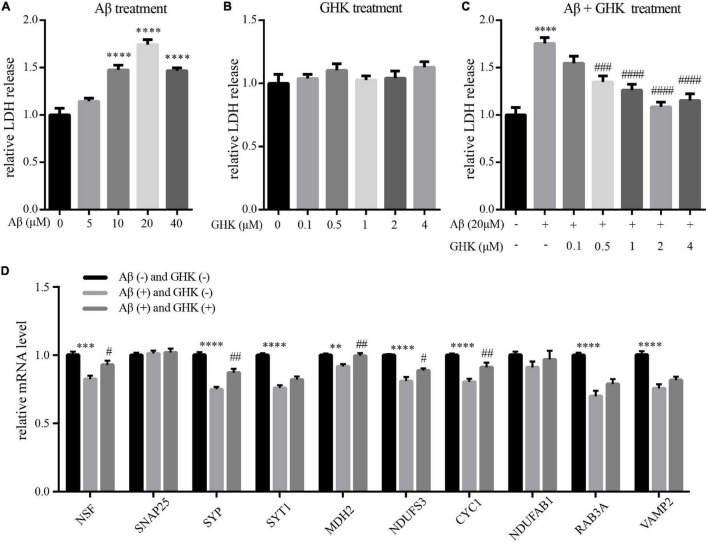
The neuroprotective effect of tripeptide GHK against Aβ_25–35_-induced cytotoxicity in differentiated human SH-SY5Y cells. **(A–C)** Statistical data showing LDH release from cells treated with **(A)** different concentrations of Aβ_25–35_ for 24 h, **(B)** different concentration of GHK for 24 h, **(C)** different concentrations of GHK for 6 h before addition of Aβ_25–35_ (20 μM) for another 24 h. **(D)** mRNA expression level of 10 hub genes in cells of control group, Aβ_25–35_ group, Aβ_25–35_ + GHK group. Values represent mean ± SEM (n = 9) and are normalized to the control group (black column). ANOVA with Dunnet’s *post-hoc* test was used to determine statistical significance. ^**^p < 0.01, ^***^p < 0.001, ^*⁣*⁣**^p < 0.0001, compared to control group, ^#^*p* < 0.05, ^##^*p* < 0.01, ^###^*p* < 0.001, ^####^*p* < 0.0001, compared to Aβ_25–35_ treatment group.

We next used Aβ_25–35_-induced neurotoxicity in differentiated human neuroblastoma SH-SY5Y cells as an *in vitro* model of AD to verify the neuroprotective effect of GHK against AD. Differentiated SH-SY5Y cells provide a closer approximation of human neuronal cells compared to their undifferentiated state. To determine the optimal dose of Aβ_25–35_ in cytotoxicity induction, differentiated SH-SY5Y cells were treated with Aβ_25–35_ at different doses (5, 10, 20 and 40 μM) for 24 h. As shown in Figure 6A, Aβ_25–35_ induced a concentration-dependent increase in LDH release. Exposure to 20 μM Aβ_25–35_ resulted in sufficient cytotoxicity, which would be used in the subsequent experiments. Treatment with GHK showed no increase in LDH release at different doses (0.1, 0.5, 1, 2 and 4 μM) (Figure 6B), which implied that a certain concentration of GHK had no neurotoxic effect on differentiated SH-SY5Y cells.

Next, GHK was added at different doses for 6 h prior to the incubation with 20 μM Aβ_25–35_ for an additional 24 h to determine the neuroprotective effect of GHK. We found that LDH release was significantly decreased in cells pre-incubated with GHK compared with cells treated with Aβ_25–35_ alone (Figure 6C). Pretreatment with 2 μM GHK showed the most decrease in LDH release and was used to verify the ability to reverse the dysregulated hub genes identified previously.

We finally examined the effects of GHK on restoring the dysregulation of 10 hub genes using qPCR. As shown in Figure 6D, the relative expression levels of hub genes including NSF, SYP, SYT1, MDH2, NDUFS3, CYC1, RAB3A, and VAMP2 (8 out of 10) were significantly decreased in SH-SY5Y cells treated with 20 μM Aβ_25–35_ for 24 h as compared with the control group. When SH-SY5Y cells were pretreated with 2 μM GHK for 6 h before Aβ_25–35_ treatment, downregulation of NSF, SYP, MDH2, NDUFS3, and CYC1 (5 out of 10) were reversed. These findings suggest that GHK protects SH-SY5Y cells against Aβ_25–35_-induced injury and reverses part of the dysregulated network in AD.

## Discussion

In this study, we identified two modules, M3 and M8, susceptible to AD by integrating co-expressed modules across multiple cerebral regions and AD-correlated genes. The DE analysis of the microarray, bulk RNA-seq, and snRNA-seq data revealed the dysregulation of M3 and M8 across different cerebral regions in both normal aging and AD. Cell-type enrichment and DE analysis of snRNA-seq implied the extensive neuronal vulnerability of M3 and M8 in AD pathogenesis. Drug repurposing analysis prioritized GHK to probably restore the dysregulated network toward health. *In vitro* experiments further verified the neuroprotective effect of GHK and its ability to reverse the dysregulated hub genes.

Since aging is the most important risk factor for AD, a critical unsolved problem is whether the gene expression changes in the AD brain have been initiated during aging. For decades, Aβ is the primary target for exploring AD therapies, but to date, anti-Aβ approaches have not yet produced positive results in clinical trials (Ayton and Bush, 2021). The trial failures cast doubt on the validity of the Aβ hypothesis. Meanwhile, extensive research has demonstrated that the dysfunction of cellular pathways such as energy metabolism, synaptic transmission, and myelin-axon interaction appear before neuropathological changes in PFC, EC, and HIP (Blalock et al., 2004; Miller et al., 2008; Patel et al., 2019; Stefanova et al., 2019b). These studies emphasize abnormal synaptic activity and energy metabolism as early events in AD. By analyzing multiple transcriptome datasets, our study further highlights the critical roles of synapse activity and energy metabolism across multiple cerebral regions and extensive neuronal subtypes in normal aging and AD pathogenesis. Overall, our findings support the possibility that AD might be a disorder further dysregulated from normal aging across different brain regions. The dysfunctional regulatory network in extensive neuronal cells of different brain regions might be related to cognitive impairment and diverse NPS of AD.

In our study, there were a large number of genes related to synapse in AD-related modules, which were enriched in the pathways of synaptic activity, retrograde endocannabinoid signaling, ion transport, and cytoskeleton organization. Synaptic activity and plasticity are the basis of memory and cognition. Synaptic vesicles trafficking and neurotransmitters release are strictly regulated by a group of highly-conserved proteins, collectively called soluble N-ethylmaleimide-sensitive fusion protein attachment protein receptors (SNAREs) (Margiotta, 2021). We found that many SNAREs and SNARE accessory proteins such as SNAP25, VAMP2, NSF, and SYP exhibited downregulated expression in multiple brain regions of aging and AD. A previous microarray study also revealed the decrease of synaptic gene expression in multiple brain regions of aging and AD brain (Berchtold et al., 2013). The downregulation or mutation of these vesicle transport genes were found to greatly cause synaptic dysfunction and cognitive impairment (Schmitt et al., 2009; Hoerder-Suabedissen et al., 2019; Salpietro et al., 2019), which reveals the altered expression of synaptic genes linked to cognitive deficits.

Oxidative phosphorylation was another enriched pathway of DEGs in AD-related modules. Mitochondrial oxidative phosphorylation is the primary source of cellular ATP synthesis. Neuronal cells rely heavily on the high-efficiency of ATP production to maintain various basic functions, such as (a) maintaining the electrical and concentration gradients of multiple ions, which is necessary for the axon and synaptic membrane potential; (b) synaptic vesicles anchoring, releasing and recovering; (c) maintaining intracellular calcium homeostasis (Ly and Verstreken, 2006). Our results showed that multiple genes involved in oxidative phosphorylation were downregulated in AD, which might lead to decreased ATP production. ATP shortage in neurons results in disorders of neurotransmission, oxidative stress, and calcium homeostasis, and finally leads to cell death (Ly and Verstreken, 2006). In rodents, senescence-accelerated OXYS rats at the preclinical stage were reported to exhibit characteristic changes in hippocampal mitochondria, including age-dependent ultrastructural differences, mitochondrial gene expression changes, and decreased activity of several electron transport chain enzymes (Complexes I, IV, and V) (Tyumentsev et al., 2018; Stefanova et al., 2019b). A key finding was that treatment with mitochondria-targeted antioxidants reduced hippocampal Aβ protein and tau hyperphosphorylation levels (Stefanova et al., 2019a), which demonstrates a possible causal relationship between mitochondrial function and AD.

Our results of drug repositioning and *in vitro* experiments preliminarily indicated the neuroprotective effect of GHK against AD. GHK, a natural tripeptide, was found to decline rapidly with aging in human serum (Pickart and Margolina, 2018). Several *in vitro* studies indicated that GHK had multiple biological effects on cultured cells, such as stimulating angiogenesis and nerve outgrowth, increasing collagen, elastin, and glycosaminoglycan synthesis (Alberghina et al., 1992; Ahmed et al., 2005; Arul et al., 2005; Pollard et al., 2005). A notable feature of GHK is the selective and high-binding affinity toward metal ions Cu^2+^ (Pickart and Lovejoy, 1987). Multiple clinical studies have demonstrated the important role of Cu^2+^ in the pathogenesis of AD. An increased level of plasmatic Cu unbound to ceruloplasmin (nCp-Cu, also known as “free copper”) was found to be correlated with brain atrophy, CSF levels of Aβ and p-tau, and more severe clinical courses in patients with AD (Squitti et al., 2006; Strozyk et al., 2009; Diouf et al., 2020). Abnormal interaction between Aβ peptides and Cu^2+^ promoted Aβ aggregation and neurotoxicity (Zou et al., 2001; Bellingham et al., 2004; Innocenti et al., 2010). However, it is interesting that DEGs between GHK treatment and vehicle were not enriched in copper metabolism or Aβ-related pathways. Together with our functional experiments of GHK reversing the downregulation of hub genes, we, for the first time, verify that GHK also provides a neuroprotective effect through regulating cellular metabolism and synaptic activity besides Cu^2+^ metabolism. In addition to GHK, previous studies have discovered other tripeptides exhibiting neuroprotective effects against AD. KED (Lys-Glu-Asp) (Khavinson et al., 2021), EDR (Glu-Asp-Arg) (Khavinson et al., 2020), and GPE (Gly-Pro-Glu) (Turkez et al., 2021) were found to prevent dendritic spines loss and neuroplasticity impairments in both *in vitro* and *in vivo* models of AD although they have different amino acid compositions. Compared with other tripeptides, GHK was screened by its ability to restore the dysregulated co-expression network of LOAD, which might be more conducive to clinical translation. These studies consistently suggest that tripeptides can act as modulators of multiple cellular pathways involved in AD pathogenesis. Therefore, peptide bioregulators appear to be a group of potential drug candidates for the treatment of AD.

The blood-brain barrier (BBB) penetration capability of therapeutic agents is a crucial problem in the central nervous system drug discovery process, but there is currently no study about the ability of GHK to penetrate the BBB. Recent evidence suggests that short peptides may enter the brain through carrier-mediated transport, receptor-mediated transcytosis, or adsorptive-mediated transcytosis (Zhou et al., 2021). Whether GHK can cross the BBB through these approaches requires further investigations. If not, drug delivery systems (DDS) can be considered for delivering short peptides across the BBB, such as polymeric nanoparticles, liposome, metal-based nanoparticles, and cyclodextrins. Xiao et al. (2016) previously designed a new nanomaterial, graphene quantum dots (GQDs) conjugated neuroprotective peptide Gly-Pro-Glu and found that it could improve the learning and memory capability of APP/PS1 transgenic mice by intravenous injection.

Our study also has some limitations. Firstly, CMap collects transcriptional profiles from human cell lines treated with small-molecule compounds and we cannot exclude the possibility that restoration of dysregulated gene expression after being treated with drug candidates may not apply to human disease tissues, especially in non-cancerous diseases. Even so, Cmap has been proved useful in predicting potential drug candidates for subsequent experimental validation in some brain diseases including Parkinson’s disease (Gao et al., 2014), ischemic stroke (Luo et al., 2019), and perioperative neurocognitive disorder (Wu et al., 2021). Secondly, since GHK was predicted by network-based drug discovery, we cannot exclude the possibility that GHK may show reactivity to additional off-targets besides primary targets, perturb unintended signaling pathways and finally cause side effects. Thus, it is critical to explore whether GHK affects the activity of other pathways. The application of network-based approaches to predict computational drug-target interaction networks (DTNs) can identify the potential molecular pathways affected by GHK (Agamah et al., 2020). High-throughput sequencing technologies such as transcriptome, proteome, and metabolome sequencing also provide potent approaches to describe the comprehensive map of molecular targets of GHK (Dai and Zhao, 2015). Thirdly, our experimental results suggested that pretreatment with GHK protected against Aβ-induced injury, but only reversed half of the dysregulated gene networks in differentiated SH-SY5Y cells, possibly because this *in vitro* model cannot simulate the whole pathological processes of AD. Human-induced pluripotent stem cell (hiPSC)-derived neuronal cells from AD patients might be a better choice for functional experiments validation (MacDougall et al., 2021). On the other hand, even though transgenic and senescence-accelerated mouse models cannot perfectly simulate the pathogenesis of LOAD, it is still valuable to explore the ameliorating effect and potential side effects of GHK in *in vivo* research.

## Data Availability Statement

The datasets presented in this study can be found in online repositories. The names of the repository/repositories and accession number(s) can be found in the article/Supplementary Material.

## Author Contributions

HZ and ZW conceived and designed the study. JZ and QL performed the bioinformatics analysis and carried out the experiments with assistance from WW, XZ, ZZ, and YL. JZ wrote the manuscript. All authors revised and approved the final manuscript.

## Conflict of Interest

The authors declare that the research was conducted in the absence of any commercial or financial relationships that could be construed as a potential conflict of interest.

## Publisher’s Note

All claims expressed in this article are solely those of the authors and do not necessarily represent those of their affiliated organizations, or those of the publisher, the editors and the reviewers. Any product that may be evaluated in this article, or claim that may be made by its manufacturer, is not guaranteed or endorsed by the publisher.

## Footnotes

1
https://www.ncbi.nlm.nih.gov/geo/

2
https://www.gtexportal.org/

3
https://www.synapse.org/

4
http://metascape.org/gp/

5
https://string-db.org/

6
https://portals.broadinstitute.org/cmap/

7
https://www.r-project.org
