# Supplementary material for: Discovery of Novel Drug Candidates for Alzheimer’s Disease by Molecular Network Modeling
Source: Front Aging Neurosci. 2022 Apr 15;14:850217. doi: 10.3389/fnagi.2022.850217 (PMC9051440; doi:10.3389/fnagi.2022.850217)
Supplement: Supplementary file 6 [file Data_Sheet_1.docx]

Supplementary Material

# Supplementary Figures and Tables

## Supplementary Figures


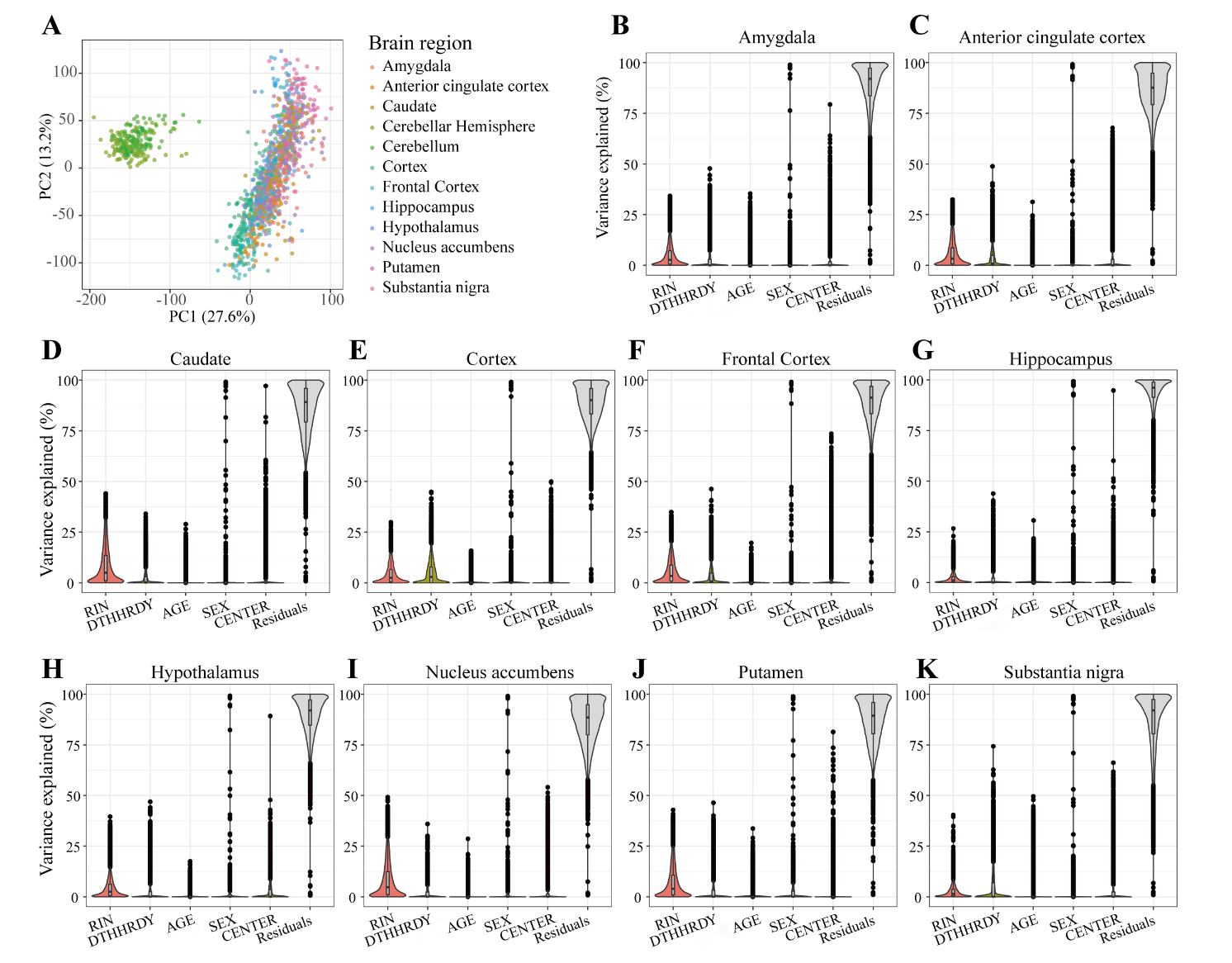


**Supplementary Figure 1.** **(A)** PCA scatter plot of gene expression data across 12 brain regions. **(B-K)** Percentage of variance explained by age, gender, RIN, center, and DTHHRDY for each gene of 10 cerebral regions.


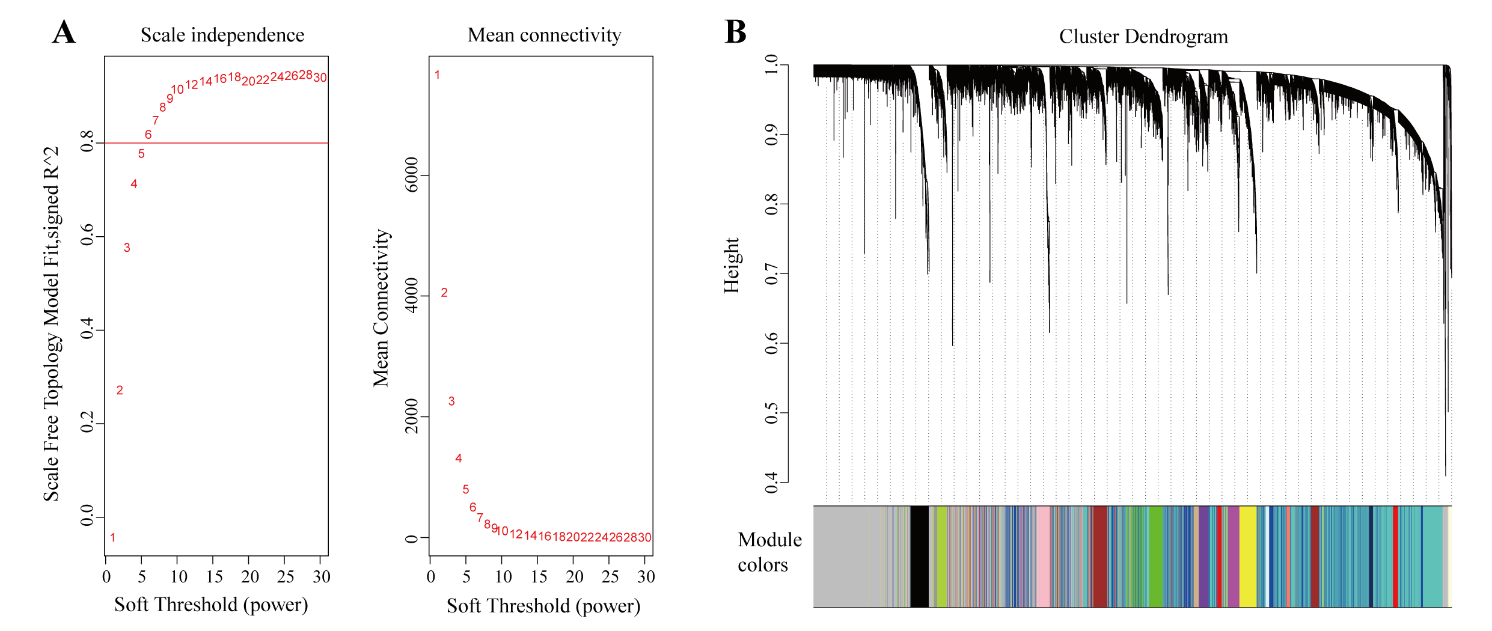


**Supplementary Figure 2.** Co-expression modules construction across 10 cerebral regions using WGCNA. **(A)** Plot of scale-free fit index and mean connectivity for various soft-thresholding powers in WGCNA. **(B)** Hierarchical clustering dendrograms of modules with corresponding color assignments. The gray module consists of genes not assigned to any module.


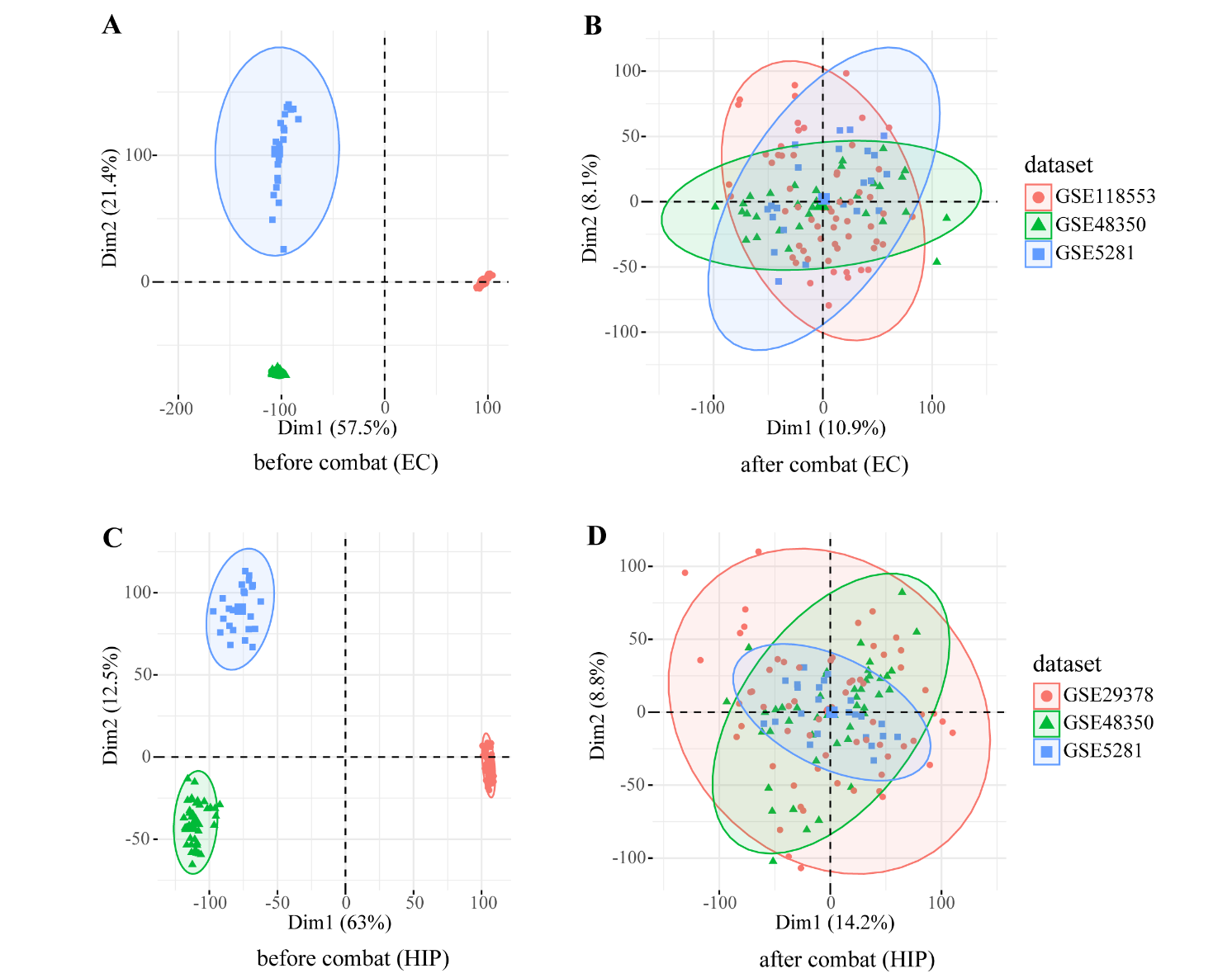


**Supplementary Figure 3.** PCA plot before and after ComBat batch effect removal. Different datasets of EC **(A, B)** and HIP **(C, D)** used for batch correction are shown on this figure. EC: entorhinal cortex, HIP: hippocampus.


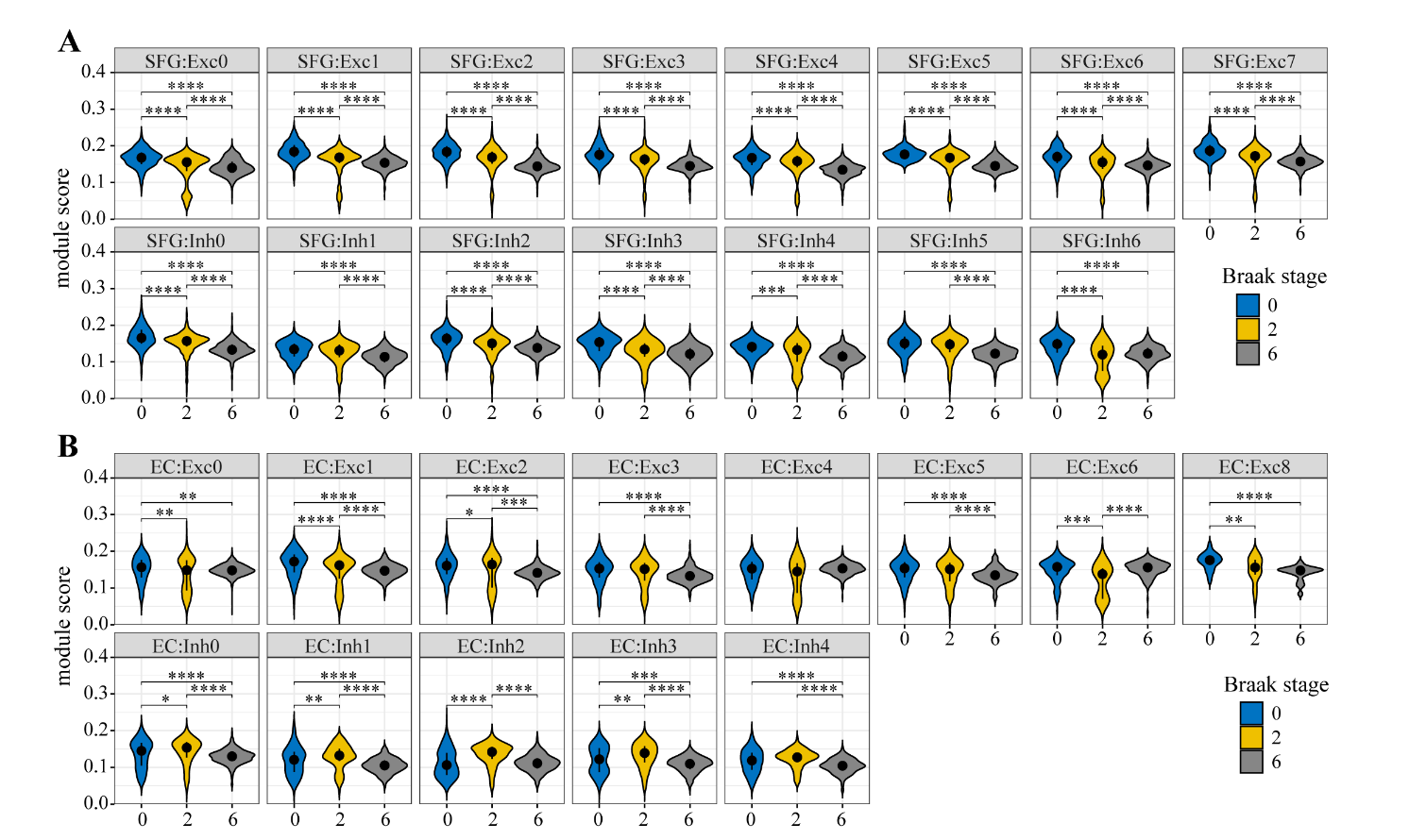


**Supplementary Figure 4.** Relative expression level of M3 and M8 between different Braak stages in different neuronal subtypes of SFG **(A)** and EC **(B)**. Kruskal-Wallis test with Benjamini-Hochberg correction was used to determine statistical significance. The black circle and bar indicate median and quartiles (25th and 75th percentile), respectively. * q < 0.05, ** q < 0.01, *** q < 0.001, **** q < 0.0001. SFG: superior frontal gyrus; EC: entorhinal cortex; Exc: excitatory neurons; Inh: inhibitory neurons.

## Supplementary Tables

**Supplementary Table 1.** Expression datasets used in this study.

**Supplementary Table 2.** Sequences of primer pairs used for RT-PCR in this study.

**Supplementary Table 3.** AD-related genes collected from two public datasets and one review.

**Supplementary Table 4.** List of DEGs with absolute logFC > 0.2 and q < 0.05 in at least four brain regions.

**Supplementary Table 5.** GSEA enrichment pathways of GHK-induced transcriptional changes.
